# Patient-Reported Outcomes of Nirmatrelvir Treatment for High-Risk, Nonhospitalized Adults With Symptomatic COVID-19

**DOI:** 10.1093/ofid/ofaf449

**Published:** 2025-08-01

**Authors:** Wajeeha Ansari, Henriette Coetzer, Kelly A Gebo, Jinma Ren, Amie Scott, Joseph C Cappelleri, Ashley S Cha-Silva, Heidi Leister-Tebbe

**Affiliations:** Global Biopharmaceuticals Group, Pfizer Inc., New York, New York, USA; Blue Health Intelligence® (BHI®), Chicago, Illinois, USA; Johns Hopkins University School of Medicine, Baltimore, Maryland, USA; Global Biometrics and Data Management, Pfizer Inc., Collegeville, Pennsylvania, USA; Global Real World Evidence, Pfizer Inc., New York, New York, USA; Global Biometrics and Data Management, Pfizer Inc., Groton, Connecticut, USA; Global Biopharmaceuticals Group, Pfizer Inc., New York, New York, USA; Pfizer Research and Development, Pfizer Inc., Collegeville, Pennsylvania, USA

**Keywords:** antiviral agents, COVID-19/drug therapy, health-related quality of life, patient outcomes, viral protease inhibitors

## Abstract

**Background:**

Nirmatrelvir/ritonavir (NMV/r) is an antiviral treatment for COVID-19. We describe patient-reported outcomes with NMV/r in nonhospitalized adults with COVID-19 at high risk of severe disease.

**Methods:**

In a phase 2/3 double-blind study, high-risk adults with SARS-CoV-2 and ≤5 days of symptoms were randomized 1:1 to receive NMV/r or placebo twice daily for 5 days. Patients recorded responses to 3 Global Impression Questions (GIQs) daily through day 28 to assess return to usual health, return to usual activities, and overall severity of COVID-19-related symptoms. The COVID-19-specific Work Productivity and Activity Impairment Questionnaire (WPAI-COVID-19) and EuroQol Quality of Life 5-dimension 5-level scale (EQ-5D-5L) were completed at specified visits through week 24.

**Results:**

Compared with placebo, patients receiving NMV/r had a significant 3-day reduction in median time to return to usual health (hazard ratio [HR], 1.3; 95% confidence interval [CI], 1.2‒1.4; *P* < .0001), 1-day reduction in median time to return to usual activities (HR, 1.2; 95% CI, 1.1‒1.4; *P* < .0001), and significantly shorter GIQ times to sustained resolution of any overall symptoms (HR, 1.2; 95% CI, 1.1‒1.3; *P* = .0002) and sustained alleviation of any overall symptoms (HR, 1.2; 95% CI, 1.1‒1.3; *P* < .0001). There were no significant differences between treatment groups in the WPAI-COVID-19 or EQ-5D-5L results.

**Conclusions:**

Patients receiving NMV/r for COVID-19 reported a reduced duration and severity of COVID-19 symptoms based on their global impression of overall symptom burden and quicker return to usual health and usual activities compared with those receiving placebo.

**Clinical Trial Information:**

ClinicalTrials.gov, NCT04960202, https://clinicaltrials.gov/study/NCT04960202

## Teaser

In a phase 2/3 trial, high-risk adults with confirmed symptomatic COVID-19 receiving nirmatrelvir/ritonavir twice daily for 5 days reported global impressions of reduced symptom severity, quicker symptom resolution, and reduced likelihood of worsening symptoms compared with patients receiving placebo.

COVID-19 poses a continuing burden to public health and healthcare systems [1, 2]. Like the early days of the pandemic, the risk of developing severe disease remains greatest among older individuals and those with underlying clinical conditions, such as cardiovascular disease, respiratory disease, diabetes, and obesity [3, 4].

In addition to the physical health consequences of COVID-19, affected patients report a substantial impact of the disease on their quality of life [5–8]. Across multiple reports from several countries, researchers have identified significant reductions in health-related quality of life (HRQoL) and productivity associated with acute COVID-19 (within ≤4 weeks of diagnosis) and after acute COVID-19, or postacute sequelae of SARS-CoV-2 infection (generally considered as symptoms lasting at least 2 months beyond initial infection) [6, 7, 9]. Impairments spanned many HRQoL domains, including mood, activities associated with daily living, and social functioning [5, 7]. A significantly greater burden was observed among women, older patients, patients from low- or middle-income countries or who had lower health literacy, patients with comorbidities, and patients with more severe acute disease [5, 6].

Nirmatrelvir is a potent, selective SARS-CoV-2 main protease inhibitor orally administered along with ritonavir (nirmatrelvir/ritonavir [NMV/r]; Paxlovid) to enhance the pharmacokinetic profile [10]. The US Food and Drug Administration (FDA) granted NMV/r Emergency Use Authorization in December 2021 for individuals aged ≥12 years and approval on 25 May 2023, for adults for the treatment of mild-to-moderate COVID-19 among those at high risk of progression to severe disease [11–13]. Approval was based primarily on data from the pivotal phase 2/3 study, which demonstrated an 86% reduction in risk of COVID-19-related hospitalization or all-cause death within 28 days among high-risk patients randomized to receive NMV/r versus placebo [11, 14]. Clinical results have since been corroborated by reports of real-world effectiveness of NMV/r among patients with COVID-19 worldwide [15–21]. NMV/r treatment has also been shown to reduce the duration of COVID-19-related signs and symptoms and patient healthcare utilization [22]. However, the effects of NMV/r on patient-reported experiences of work productivity, daily activities, and HRQoL have not yet been published.

In the pivotal EPIC-HR (Evaluation of Protease Inhibition for COVID-19 in High-Risk Patients) study investigating the efficacy and safety of NMV/r among patients with COVID-19 at high risk of progression to severe disease, patient-reported outcomes (PROs) were also collected to evaluate treatment impact on patient impressions of their symptom burden, work productivity, global impression of health, and overall HRQoL following COVID-19 diagnosis. Here, we report results from patient-reported global assessments of health using the Global Impression Questions (GIQs), COVID-19-specific Work Productivity and Activity Impairment (WPAI-COVID-19), and EuroQol Quality-of-Life 5-dimension 5-level scale (EQ-5D-5L) questionnaires administered during the clinical study.

## METHODS

### Clinical Study Design

Detailed methods of the pivotal EPIC-HR study were reported previously [14]. Briefly, nonhospitalized unvaccinated adults at high risk of progressing to severe disease with confirmed symptomatic COVID-19 were randomized 1:1 within 5 days of symptom onset to receive either NMV/r treatment or placebo twice daily for 5 days. The primary endpoint was the proportion of patients experiencing COVID-19-related hospitalization or all-cause death through day 28 for those whose treatment started within 3 days after onset of COVID-19 symptoms [14]. Key secondary endpoints included the comparison evaluated similarly for patients whose treatment started within 5 days after onset of COVID-19 symptoms, time (days) to sustained alleviation, and time (days) to resolution of all targeted symptoms through day 28 [14, 22]. In this analysis, PROs were collected as other endpoints during the main study and long-term follow-up period through week 24 and not considered primary or secondary study endpoints. PROs were evaluated in all randomized patients who received ≥1 dose of their assigned treatment and analyzed according to the study intervention to which they were randomized.

All procedures were in accordance with ethical guidelines defined in the Declaration of Helsinki of the World Medical Association.

### Collection of PROs

Patients were asked to complete 3 questionnaires throughout the study period: the GIQ, WPAI-COVID-19, and EQ-5D-5L. Responses were collected via an electronic diary, referred to as an ePRO, and assessed only in patients who enrolled after an initial cohort of 60 patients were enrolled; thus, responses were available from a subset of participants.

The GIQ [23] consisted of 3 questions that collected patient impressions of their return to usual health, return to usual activities, and overall assessment of COVID-19-related symptoms (Supplementary Table 1). Time (days) to return to usual health or usual activities was defined as the number of days from receipt of the first dose until the first day that the patient returned to usual health or activities, plus 1 day. We calculated time (days) to sustained resolution of GIQ moderate-to-severe overall COVID-19 symptoms and to sustained resolution of any GIQ overall symptoms (mild, moderate, and severe). We calculated time (days) to sustained alleviation of any GIQ overall COVID-19 symptoms. Missing severity at baseline was treated as mild. The GIQ sustained resolution of overall symptoms was defined a priori as achieving 4 consecutive days with no reported overall symptoms. The GIQ sustained alleviation of overall symptoms was defined as (1) achieving 4 consecutive days of mild or absent symptoms after reporting moderate or severe symptoms at baseline or (2) achieving 4 consecutive days with no reported symptoms after reporting mild symptoms at baseline. The time to sustained resolution or alleviation of GIQ overall COVID-19 symptoms was calculated as the first event date minus the first dose date, plus 1 day. Patients were asked to complete the GIQ by ePRO at baseline and each day for 28 days.

The WPAI-COVID-19 was adapted from the General Health version of the WPAI [24] to evaluate changes over time in the impact of COVID-19 on absenteeism, presenteeism, and overall productivity. It consists of 6 questions regarding current employment status, hours missed due to health problems, hours missed for other reasons, hours worked, and the extent to which work productivity and other unpaid activities were affected by health (using a 0‒10 scale) during the past 7 days (Supplementary Table 1). Patients completed the WPAI-COVID-19 by ePRO on days 5 and 14 and weeks 12 and 24 as the instrument became available.

The EQ-5D-5L, a standardized and generic HRQoL questionnaire, is used to evaluate mobility, self-care, typical activities, pain and discomfort, anxiety and depression, and health status on the day of questionnaire completion by incorporating both descriptive questions (using preference weights from the United Kingdom to generate an index score from −1 to 1) and a visual analog scale (VAS; generating a VAS score from 0 to 100); on both scales, higher scores represent better health (Supplementary Table 1) [25]. The EQ-5D-5L was completed by patients by ePRO at baseline; on days 5, 14, and 34; and at weeks 12 and 24 as the instrument became available.

The 3 GIQ questions were collected in the ePRO of each enrolled participant from the study start. Implementation of the EQ-5D-5L and WPAI-COVID-19 in the ePRO commenced after the initiation of data collection for the primary efficacy outcome and daily symptom diary for key secondary outcomes because of operational constraints, including ePRO application integration, institutional review board review schedules, and execution of 27 language translations. Consequently, there is variation in participant numbers between baseline and follow-up assessments within the EQ-5D-5L and WPAI-COVID-19 analyses, as well as when compared with the overall trial population.

### Statistical Analysis

All PRO analyses included participants randomly assigned to a study intervention who received ≥1 dose of the study intervention and had ≥1 postbaseline visit through day 28 (modified intent-to-treat 2 [mITT2] population). Time to return to usual health and usual activities, time to sustained resolution of moderate or severe symptoms, time to sustained resolution or alleviation of any symptoms, and time to progression to increased disease severity derived from the GIQ were analyzed using the Kaplan–Meier method [26] with a log-rank test [27] and a Cox proportional hazard model [28] to calculate hazard ratios (HRs) with 95% confidence intervals (CIs) to compare differences between the 2 treatment groups [29]. In the Cox proportional hazard analysis, treatment group (NMV/r or placebo) and geographic region were used as independent variables, and controlled covariates included symptom onset duration (≤3 or >3 days), COVID-19 monoclonal antibody treatment (yes or no), baseline SARS-CoV-2 serology status (positive or negative), and baseline viral load (<4 or ≥4 log_10_ copies/mL). For the Cox proportional hazard analysis, the proportional hazard assumption was evaluated using graphical diagnostics.

For the WPAI-COVID-19 and EQ-5D-5L questionnaires, completion status was summarized for each time point. Data collected from the WPAI-COVID-19 and EQ-5D-5L were analyzed using a mixed-effects model with repeated measures (MMRM) [30] adjusting for the status of baseline PRO measurements (missing or not), treatment, time (days from baseline), and treatment-by-time interaction. The model included all available baseline and postbaseline measurements. Descriptive WPAI-COVID-19 and EQ-5D-5L results were presented for the overall population.

Statistical analyses were conducted using SAS version 9.4 (Cary, North Carolina, United States).

## RESULTS

### Patient Population

**Figure 1.**
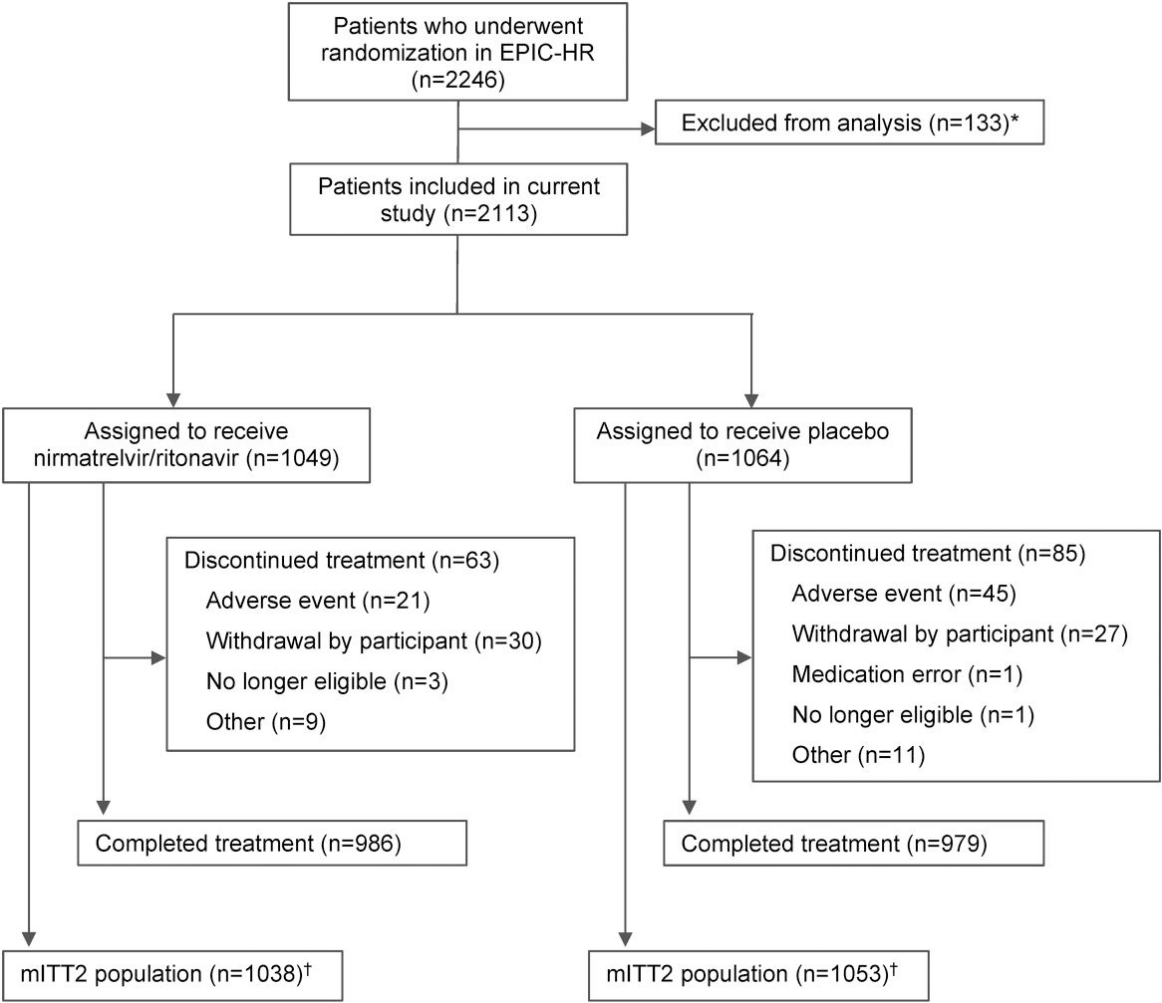
CONSORT diagram detailing enrollment and randomization. *Data were excluded from 2 sites from the full analysis set due to data quality issues. ^†^mITT2 population includes all patients who received ≥1 dose of the study intervention and had ≥1 postbaseline visit through day 28. mITT2 , modified intent-to-treat 2.

From the 2246 randomized study participants, the mITT2 population for evaluation included 2091 patients (NMV/r = 1038, placebo = 1053; Figure 1). Demographic and baseline clinical characteristics were similar between the 2 treatment groups in the mITT2 population (Table 1).

**Table 1.** Patient Demographic and Baseline Clinical Characteristics of the mITT2 Population

Characteristic	Nirmatrelvir/Ritonavir 300 mg/100 mg (N = 1038)	Placebo (N = 1053)	Total (N = 2091)
Median (range) age, y	44.0 (18.0–86.0)	46.0 (18.0–88.0)	45.0 (18.0–88.0)
Male, n (%)	516 (49.7)	538 (51.1)	1054 (50.4)
Race, n (%)			
White	728 (70.1)	756 (71.8)	1484 (71.0)
Black or African American	52 (5.0)	35 (3.3)	87 (4.2)
Asian	153 (14.7)	156 (14.8)	309 (14.8)
American Indian or Alaska Native	95 (9.2)	94 (8.9)	189 (9.0)
Not reported	8 (0.8)	9 (0.9)	17 (0.8)
BMI, mean (SD), kg/m^2^	29.0 (5.4)	29.2 (5.7)	29.1 (5.5)
Geographic region, n (%)			
United States	387 (37.3)	399 (37.9)	786 (37.6)
Europe	330 (31.8)	333 (31.6)	663 (31.7)
India	94 (9.1)	97 (9.2)	191 (9.1)
Rest of world	227 (21.9)	224 (21.3)	451 (21.6)
Mean (SD) duration since first diagnosis, days	1.2 (1.3)	1.3 (1.2)	1.2 (1.2)
Mean (SD) duration since first symptom, days^a^	2.9 (1.1)	3.0 (1.1)	2.9 (1.1)
Number of risk factors of interest, n (%)^b^			
1	437 (41.8)	406 (38.6)	840 (40.2)
2	364 (35.1)	381 (36.2)	745 (35.5)
3	161 (15.5)	179 (17.0)	340 (16.3)
≥4	78 (7.6)	87 (8.3)	165 (7.9)
SARS-CoV-2 serology status, n (%)			
Negative	504 (48.6)	526 (50.0)	1030 (49.3)
Positive	521 (50.2)	514 (48.8)	1035 (49.5)
Unknown	13 (1.3)	13 (1.2)	26 (1.2)
Baseline viral load, mean (SD), log_10_ copies/mL	4.8 (2.9)	4.7 (2.9)	4.7 (2.9)

Abbreviations: BMI, body mass index; mITT2, modified intent-to-treat 2; SD, standard deviation.

^a^Days from first symptom onset until enrollment.

^b^Risk factors included age ≥60 y, BMI >25; and medical history mentioning cigarette smoker, immunosuppression, chronic kidney disease, hypertension, diabetes mellitus, cardiovascular disorder, chronic lung disease, HIV infection, sickle cell disease, neurodevelopmental disorder, cancer, and/or device dependence.

### Global Impressions Questions

**Figure 2.**
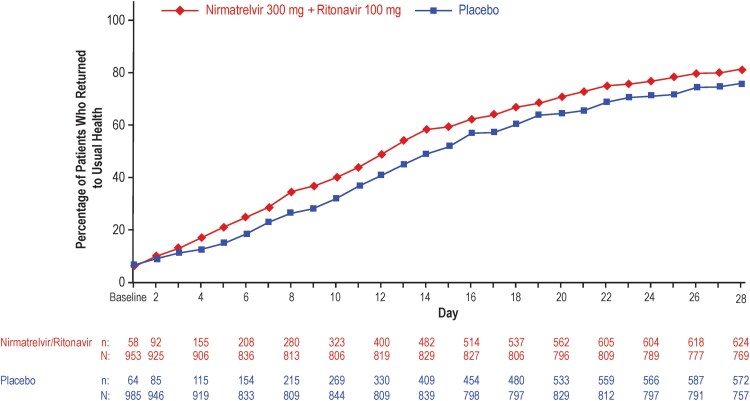
Percentage of participants in each treatment group in the mITT2 population reporting return to usual health on each study day through day 28 on the GIQ. mITT2, modified intent-to-treat 2; n, number of patients with event; N, number of patients with nonmissing data.

Reductions in time to return to usual health and usual activities, as well as global impression of overall COVID-19 symptom burden, were observed across all measures of the GIQ among patients who received NMV/r compared with placebo. Of patients who completed the GIQ on day 28, more who had received NMV/r reported having returned to their usual health status at day 28 compared with placebo (81% [624/769] vs 76% [572/757]). Differences between the 2 groups emerged as early as day 4 (Figure 2). Patients who received NMV/r reported a quicker median time to return to usual health (10 days; 95% CI, 10‒11 days) by 3 days compared with placebo (13 days; 95% CI, 12‒14 days; HR: 1.27; 95% CI, 1.2‒1.4; *P* < .0001).

**Figure 3.**
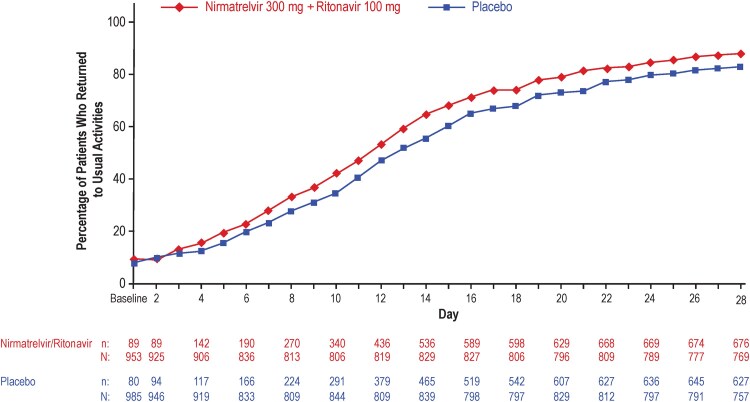
Percentage of participants in each treatment group in the mITT2 population reporting return to usual activities on each study day through day 28 on the GIQ. mITT2, modified intent-to-treat 2; n, number of patients with event; N, number of patients with nonmissing data.

A return to usual activities was reported by 88% (676/769) versus 83% (627/757) of patients in the NMV/r and placebo groups by day 28 (Figure 3). Patients who received NMV/r reported a 1-day reduction in median time to return to usual activities compared with placebo (11 days; 95% CI, 10‒11 days vs 12 days; 95% CI, 11‒12 days; HR: 1.2; 95% CI, 1.1‒1.4; *P* < .0001).

**Figure 4.**
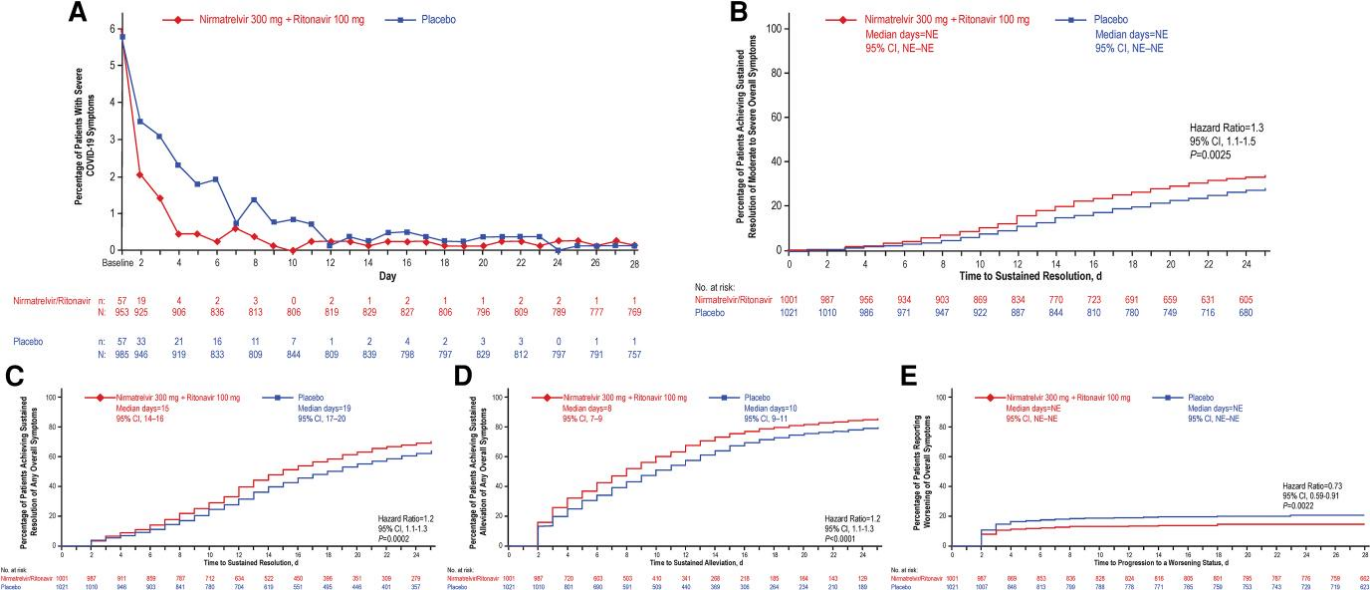
Patient perceptions of daily COVID-19 symptom severity for 28 d according to the GIQ in the mITT2 population. (*A*,) Percentage of patients with severe COVID-19 symptoms on the GIQ and Kaplan–Meier analyses showing (*B*) time to achieving sustained resolution of moderate-to-severe overall symptoms on the GIQ, (*C*) time to achieving sustained resolution of any overall symptoms on the GIQ, (*D*) time to achieving sustained alleviation of any overall symptoms on the GIQ, and (*E*) time to progression to worsening of symptoms on the GIQ. GIQ, Global Impressions Questionnaire; mITT2, modified intent-to-treat 2; n, number of patients with event; N, number of patients with nonmissing data; NE, not estimable.

The GIQ also asked patients to describe their impressions of the overall severity of their COVID-19 symptoms from baseline through day 28. By day 3, 1.4% (13/922) of patients in the NMV/r group and 3.1% (29/938) in the placebo group reported their global impression of their overall COVID-19 symptoms as severe, and differences between groups persisted until day 12 (Figure 4*A*). Similarly low proportions of patients in each group reported an impression that their overall COVID-19 symptoms were severe through day 28. Compared with patients in the placebo group, patients receiving NMV/r reported significantly reduced time to achieving sustained resolution of moderate-to-severe overall symptoms (HR, 1.3; 95% CI, 1.1‒1.5; *P* = .0025), sustained resolution of any overall symptoms (HR, 1.2; 95% CI, 1.1‒1.3; *P* < .0002), and sustained alleviation of any overall symptoms (HR, 1.2; 95% CI, 1.1‒1.3; *P* < .0001; Figure 4*B*‒*D*). Risk of progressing to increased symptom severity was significantly lower among patients receiving NMV/r versus placebo (HR, 0.7; 95% CI, 0.6‒0.9; *P* = .0022; Figure 4*E*). For all GIQ endpoints, results were similar for the modified intent-to-treat 1 population (participants randomly assigned to, and received ≥1 dose of, study intervention, with ≥1 postbaseline visit through day 28, and at baseline did not receive nor were expected to receive COVID-19 therapeutic monoclonal antibody treatment [31]; Supplementary Figures 1–3).

### Work Productivity and Activity Impairment

Low percentages of participants completed the WPAI-COVID-19 because of operational challenges. Still, completion increased over time as the ePRO was implemented. Data were collected from 53 patients (2.5%) on day 5 (baseline for the WPAI-COVID-19), 101 patients (4.8%) on day 14, 423 patients (20.2%) at week 12, and 873 patients (41.8%) at week 24. Since few participants completed the WPAI-COVID-19 at baseline, our analysis focused only on postbaseline assessments and not change from baseline. Throughout the study, approximately half of patients reported being currently employed (ranging from 40% to 61% across days and treatment groups), with similar employment rates observed between those who received NMV/r versus placebo.

Analyses of percentages of work time missed, impairment while working, overall work impairment (owing to missed work time in combination with impairment while working), and activity impairment due to COVID-19 did not reveal any clear differences between treatment groups (Supplementary Table 2). No significant differences were observed in the MMRM comparing results from the 2 treatment groups at each postbaseline time point except for a lower percentage of overall work impairment due to COVID-19 at week 24 among those who received NMV/r (least-squares mean difference: −4.4; 95% CI, −8.4 to 0.3; *P* = .04; Supplementary Table 2).

### EuroQol Quality-of-Life 5-Dimension 5-Level Questionnaire

Like the WPAI-COVID-19, EQ-5D-5L completion was low but increased over time as the ePRO was implemented. Questionnaires were completed by 51 patients (2.4%) at baseline, 57 patients (2.7%) on day 5, 103 patients (4.9%) on day 14, 195 patients (9.3%) on day 34, 428 patients (20.5%) at week 12, and 878 patients (42.0%) at week 24. There were no statistically significant differences in baseline health overall between the NMV/r and placebo groups, with respective percentages of 11.1% and 8.3% reporting any problems with mobility, 11.1% and 4.2% reporting any problems with self-care, 22.2% and 29.1% reporting any anxiety or depression, 37.0% and 50.0% reporting any problems with usual activities, and 59.3% and 70.8% reporting any pain or discomfort. There were no statistically significant differences in the EQ-5D-5L index or VAS scores between treatment groups at all time points (Supplementary Table 3).

## DISCUSSION

In this analysis of the GIQ, EQ-5D-5L, and WPAI-COVID-19 PROs collected during the pivotal phase 2/3 EPIC-HR study evaluating NMV/r for the treatment of COVID-19 in symptomatic, nonhospitalized adults at high risk for severe disease, results from the GIQ demonstrate that patients receiving NMV/r assessed their overall impression of COVID-19 symptom severity to be less severe through day 11 compared with patients receiving placebo. Patients receiving NMV/r had quicker resolution of overall impression of COVID-19 symptoms and were less likely to experience their symptoms worsening compared with patients receiving placebo based on their GIQ assessment of overall symptom severity.

The benefits of NMV/r treatment on a patient's impression of overall symptom severity reported in this exploratory analysis of GIQ results are consistent with previously published results regarding the patient-reported presence and severity of each prespecified symptom [22]. Compared with placebo, NMV/r treatment previously demonstrated a significant 2-day reduction in the time to alleviation of all targeted COVID-19 symptoms [22], similar to the significant 2-day reduction in the time to alleviation of GIQ overall impression of COVID-19 symptoms reported here. NMV/r treatment also demonstrated a significant 3-day reduction in the time to resolution of all targeted COVID-19 symptoms [22], while a significant 4-day reduction in the time to resolution of GIQ overall impression of COVID-19 symptoms was observed in the present study. Current GIQ results, which demonstrate patient-reported reductions in overall impression of COVID-19 symptom severity and time to symptom resolution and return to usual health and usual activities, further demonstrate the clinical efficacy of NMV/r in the treatment of COVID-19 in nonhospitalized, symptomatic high-risk adults observed in the pivotal phase 2/3 study [14]. Compared with the placebo group, patients who received NMV/r had an 86% relative risk reduction in COVID-19-related hospitalization or all-cause death after 28 days and a 10-fold decrease in viral load after 5 days [12]. The results of these exploratory analyses of patient self-reported global impressions of health further demonstrate the efficacy of NMV/r.

The relationship between GIQ outcomes reported here and the previously demonstrated benefit of NMV/r on reduced duration of FDA-prespecified COVID-19-targeted symptoms and lower healthcare utilization is uncertain [22]. Analysis of participants with SARS-CoV-2 infection in the ACTIV-2/A5401 trial demonstrated significant correlation between time to sustained resolution of all targeted symptoms for 2 days with both time to sustained resolution of overall COVID-19 symptoms and time to sustained return to health for both high- and standard-risk groups [32]. Further studies are needed to evaluate the extent of correlation between GIQ outcomes, work productivity, daily activities and symptom burden, and healthcare utilization.

The main limitation of our study was the late implementation of ePRO data collection for the WPAI-COVID-19 and EQ-5D-5L questionnaires, which limited sample size, particularly at baseline and during other early visits. Although the PRO assessments were prespecified as “other analyses” in the clinical trial protocol, the ePRO tool was rolled out after the study start because of operational constraints of the devices, and the WPAI-COVID-19 and EQ-5D-5L questionnaires were added after the GIQ. The staggered nature of ePRO questionnaire execution limited responses available for participants enrolled in EPIC-HR, particularly at baseline. As such, low completion at baseline of the WPAI-COVID-19 and EQ-5D-5L meant that no meaningful conclusions could be drawn regarding the impact of NMR/r treatment on work productivity or activity impairment as measured by the WPAI-COVID-19 or on HRQoL as measured by the EQ-5D-5L. Furthermore, subgroup analyses among demographic or clinical subgroups could not be performed, and no information was collected regarding long COVID. Another important consideration is that data for these PROs were collected among unvaccinated patients from 2021, a time when Delta was the predominant variant; therefore, results may not extrapolate more widely to periods when different variants predominated. It is notable, however, that ∼50% of participants in this study were seropositive for antibodies against SARS-CoV-2, indicating that results may be applicable to populations with high rates of immunity through either vaccination or prior infection.

In conclusion, the EPIC-HR phase 2/3 study was the first randomized controlled trial of a COVID-19 therapy that included PRO study endpoints. Patients receiving NMV/r for the treatment of COVID-19 reported global impressions of reduced overall COVID-19 symptom severity, quicker resolution of symptoms, and a reduced likelihood of worsening symptoms compared with those who received placebo. Results are consistent with the previously demonstrated efficacy of NMV/r among nonhospitalized patients with symptomatic COVID-19 who are at high risk of progression to severe disease and provide a novel assessment of the positive impact of NMV/r on patient experiences after receiving treatment for COVID-19. Further research is warranted to assess the effects of NMV/r on PROs in vaccinated patients with risk factors for severe COVID-19. Additional studies should also assess the short- and long-term effects of NMV/r treatment of COVID-19 on PROs, including persistent symptoms and HRQoL.

## Supplementary Material

ofaf449_Supplementary_Data

## Supplementary Data

Supplementary materials are available at *Open Forum Infectious Diseases* online. Consisting of data provided by the authors to benefit the reader, the posted materials are not copyedited and are the sole responsibility of the authors, so questions or comments should be addressed to the corresponding author.
