# Supplementary material for: Patient-Reported Outcomes of Nirmatrelvir Treatment for High-Risk, Nonhospitalized Adults With Symptomatic COVID-19
Source: Open Forum Infect Dis. 2025 Aug 1;12(8):ofaf449. doi: 10.1093/ofid/ofaf449 (PMC12345624; doi:10.1093/ofid/ofaf449)

Supplementary Material

Supplementary Table S1. Patient-Reported Outcome Questionnaires

Global Impressions Questionnaire

Patient-reported global impression items assessing return to usual health, return to usual activities, and overall COVID-19–related symptoms:

- In the past 24 hours, have you returned to your usual health (before your COVID-19 illness)? Yes or No
- In the past 24 hours, have you returned to your usual activities (before your COVID-19 illness)? Yes or No
- In the past 24 hours, what was the severity of your overall COVID-19–related symptoms at their worst? None, Mild, Moderate, or Severe

Work Productivity and Activity Impairment Questionnaire (COVID-19 V2.0)

The following questions ask about the effect of your COVID-19 on your ability to work and perform regular activities.

1. Are you currently employed (working for pay)? Yes or No
   If No, check "No" and skip to question 6.

2. During the past seven days, how many hours did you miss from work because of problems associated with your COVID-19?
   Include hours you missed on sick days, times you went in late, left early, etc., because of your COVID-19. Do not include time you missed to participate in this study.

3. During the past seven days, how many hours did you miss from work because of any other reason, such as vacation, holidays, time off to participate in this study?

4. During the past seven days, how many hours did you actually work? *(If "0", skip to question 6.)*

5. During the past seven days, how much did your COVID-19 affect your productivity while you were working?
   Think about days you were limited in the amount or kind of work you could do, days you accomplished less than you would like, or

days you could not do your work as carefully as usual. If COVID-19 affected your work only a little, choose a low number. Choose a high number if COVID-19 affected your work a great deal.

Consider only how much COVID-19 affected productivity while you were working.

COVID-19 had no effect on my work	0 1 2 3 4 5 6 7 8 9 10	COVID-19 completely prevented me from working

CIRCLE A NUMBER

6. During the past seven days, how much did your COVID-19 affect your ability to do your regular daily activities, other than work at a job?

By regular activities, we mean the usual activities you do, such as work around the house, shopping, childcare, exercising, studying, etc. Think about times you were limited in the amount or kind of activities you could do and times you accomplished less than you would like. If COVID-19 affected your activities only a little, choose a low number. Choose a high number if COVID-19 affected your activities a great deal.

Consider only how much COVID-19 affected your ability to do your regular daily activities, other than work at a job.

COVID-19 had no effect on my daily activities	0 1 2 3 4 5 6 7 8 9 10	COVID-19 completely prevented me from doing my daily activities

CIRCLE A NUMBER

EQ-5D-5L

Under each heading, please tick ONE box that best describes your health TODAY.

Your mobility TODAY

I have no problems walking

I have slight problems walking

I have moderate problems walking

I have severe problems walking

I am unable to walk

Your self-care TODAY

I have no problems washing or dressing myself
I have slight problems washing or dressing myself
I have moderate problems washing or dressing myself
I have severe problems washing or dressing myself
I am unable to wash or dress myself

Your usual activities TODAY *(e.g. work, study, housework, family or leisure activities)*

I have no problems doing my usual activities
I have slight problems doing my usual activities
I have moderate problems doing my usual activities
I have severe problems doing my usual activities
I am unable to doing my usual activities

Your pain / discomfort TODAY

I have no pain or discomfort
I have slight pain or discomfort
I have moderate pain or discomfort
I have severe pain or discomfort
I have extreme pain or discomfort

Your anxiety / depression TODAY

I am not anxious or depressed
I am slightly anxious or depressed

I am moderately anxious or depressed
I am severely anxious or depressed
I am extremely anxious or depressed

We would like to know how good or bad your health is TODAY. You will see a scale numbered 0 to 100.
100 means the best health you can imagine. 0 means the worst heath you can imagine.

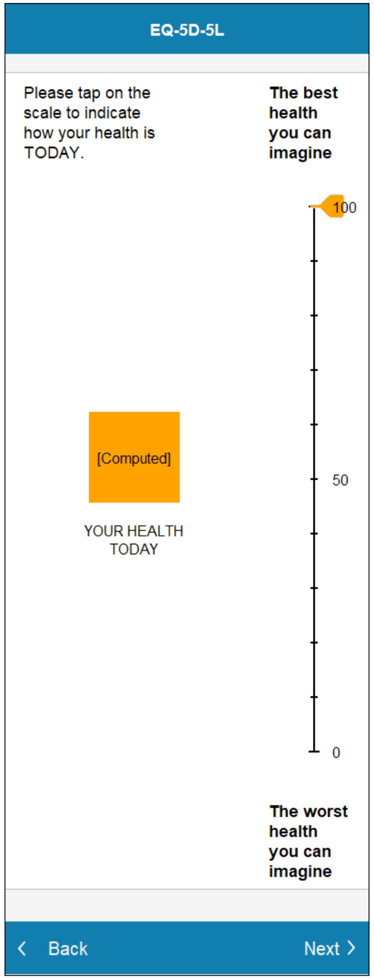
EQ-5D-5L
Please tap on the scale to indicate how your health is TODAY.
The best health you can imagine
100
50
0
[Computed]
YOUR HEALTH TODAY
The worst health you can imagine
Back
Next

-

Supplementary Table S2. Work and Activity Impairment Among Patients in the mITT2 Population Who Received Nirmatrelvir/Ritonavir or Placebo to Treat COVID-19: WPAI-COVID-19 Results (MMRM)

	Nirmatrelvir/Ritonavir N=1038		Placebo N=1053				
**Outcome**	**Respondents, n**	**LS Mean ± SE**	**Respondents, n**	**LS Mean ± SE**	**LS Mean Difference (Nirmatrelvir/Ritonavir vs Placebo)**	**95% CI**	***P* Value**
Percentage work time missed due to COVID-19							
Day 14	19	68.3±9.3	27	73.3±7.8	−5.0	−29.2, 19.1	0.68
Week 12	111	4.4±2.9	117	7.4±2.7	−3.0	−8.0, 2.1	0.25
Week 24	218	4.1±2.3	229	6.0±2.2	−2.0	−4.6, 0.7	0.15
Percentage impairment while working due to COVID-19							
Day 14	10	50.4±13.2	15	44.4±11.0	5.9	−28.5, 40.4	0.73
Week 12	111	10.4±3.9	114	8.7±3.8	1.7	−4.7, 8.1	0.60
Week 24	217	4.8±3.2	229	7.3±3.2	−2.5	−6.0, 1.0	0.16
Percentage overall work impairment due to COVID-19							
Day 14	19	84.7±8.1	27	84.8±6.9	−0.13	−20.7, 20.4	0.99

Week 12	111	15.3±3.8	117	18.3±3.6	−3.0	−9.1, 3.1	0.33
Week 24	217	8.2±3.2	229	12.6±3.1	−4.4	−8.4, −0.3	0.04
Percentage activity impairment due to COVID-19							
Day 14	46	40.5±5.2	53	44.6±4.9	−4.1	−17.5, 9.2	0.54
Week 12	198	8.9±2.7	207	10.5±2.6	−1.6	−6.8, 3.5	0.54
Week 24	425	6.1±2.2	418	5.3±2.1	0.8	−1.9, 3.5	0.57

Percentage work time missed and percentage overall work impairment due to COVID-19 were calculated among all respondents who were currently employed. Percentage work time missed due to COVID-19 was calculated among all respondents who were currently employed and who worked within the past 7 days. Percentage activity impairment due to COVID-19 was calculated among all respondents.

n=number of patients with nonmissing data in the analysis set and the covariates in the statistical model; N=number of patients in the analysis set.

LS=least squares; mITT2=modified intent-to-treat 2; MMRM=mixed-effects random effects model; SE=standard error; WPAI-COVID-19=COVID-19–specific Work Productivity and Activity Impairment Questionnaire.

Supplementary Table S3. Health-Related Quality of Life Among Patients in the mITT2 Population Who Received Nirmatrelvir/Ritonavir or Placebo to Treat COVID-19: EQ-5D-5L Results

	Nirmatrelvir/Ritonavir N=1038		Placebo N=1053				
**Score**	**Respondents, n**	**LS Mean ± SE**	**Respondents, n**	**LS Mean ± SE**	**LS Mean Difference (Nirmatrelvir/Ritonavir vs Placebo)**	**95% CI**	***P* Value**
Index score							
Day 5	28	0.83±0.03	29	0.84±0.02	−0.01	−0.07, 0.06	0.83
Day 14	47	0.92±0.02	54	0.91±0.02	0.01	−0.04, 0.06	0.76
Day 34	93	0.95±0.02	96	0.95±0.02	0.00	−0.04, 0.03	0.91
Week 12	200	0.98±0.01	210	0.96±0.01	0.02	−0.01, 0.04	0.24
Week 24	426	0.97±0.01	421	0.97±0.01	0.00	−0.02, 0.01	0.64
VAS score							
Day 5	28	71.5±3.1	28	77.4±2.9	−5.93	−13.9, 2.00	0.14
Day 14	47	83.7±2.8	54	82.4±2.0	1.39	−5.85, 8.63	0.70
Day 34	93	88.8±1.8	96	88.1±1.7	0.80	−3.16, 4.76	0.69
Week 12	200	92.1±1.3	210	91.7±1.3	0.42	−1.70, 2.55	0.69
Week 24	426	92.2±1.2	421	91.7±1.2	0.51	−1.02, 2.03	0.51

n=number of patients with nonmissing data in the analysis set and the covariates in the statistical model; N=number of patients in the analysis set.

EQ-5D-5L=EuroQol Quality-of-Life 5-dimension 5-level questionnaire; LS=least squares; mITT2=modified intent-to-treat 2; MMRM=mixed-effects random effects model; SE=standard error; VAS=visual analog scale.

Supplementary Figure 1. Modified intent-to-treat population 1: (A) percentage of participants in each treatment group reporting return to usual health on each study day through Day 28 on the Global Impressions Questionnaire and (B) Kaplan-Meier curves of return to usual health over time. The modified intent-to-treat 1 population included all participants who were randomly assigned to study intervention, received ≥1 dose of study intervention, at baseline did not receive nor were expected to receive COVID-19 therapeutic monoclonal antibody treatment, and were treated within ≤5 days of COVID-19 onset. n=number of patients with event; N=number of patients with nonmissing data.

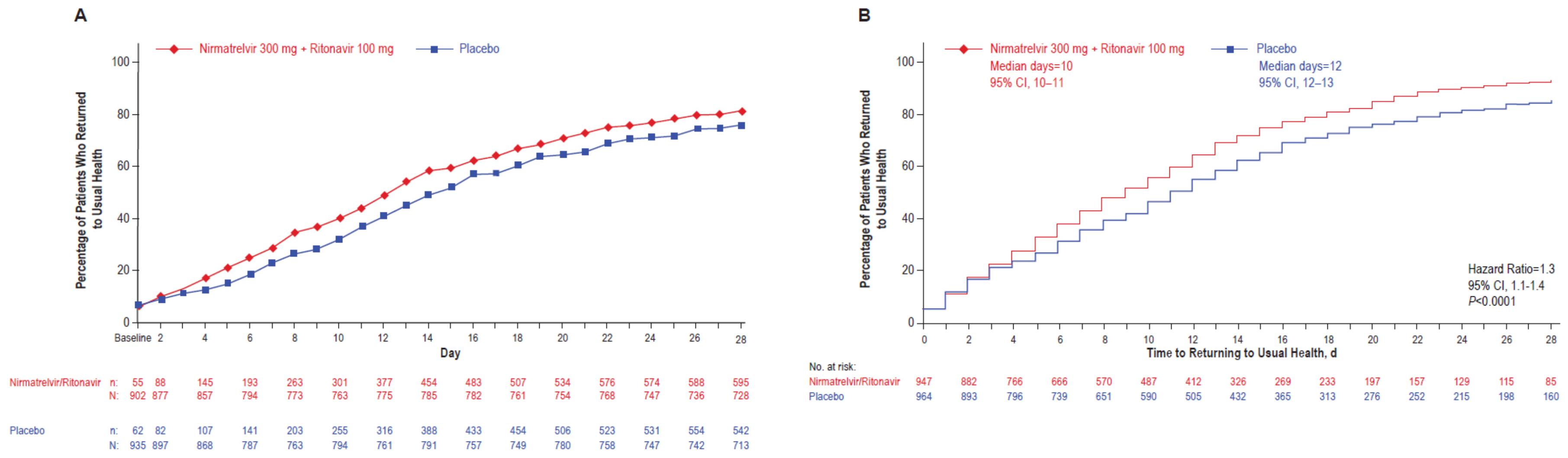

Supplementary Figure 2. Modified intent-to-treat population 1: (A) percentage of participants in each treatment group reporting return to usual activities on each study day through Day 28 on the Global Impressions Questionnaire and (B) Kaplan-Meier curves of return to usual activities over time. The modified intent-to-treat 1 population included all participants who were randomly assigned to study intervention, received ≥1 dose of study intervention, at baseline did not receive nor were expected to receive COVID-19 therapeutic monoclonal antibody treatment, and were treated within ≤5 days of COVID-19 onset. n=number of patients with event; N=number of patients with nonmissing data.

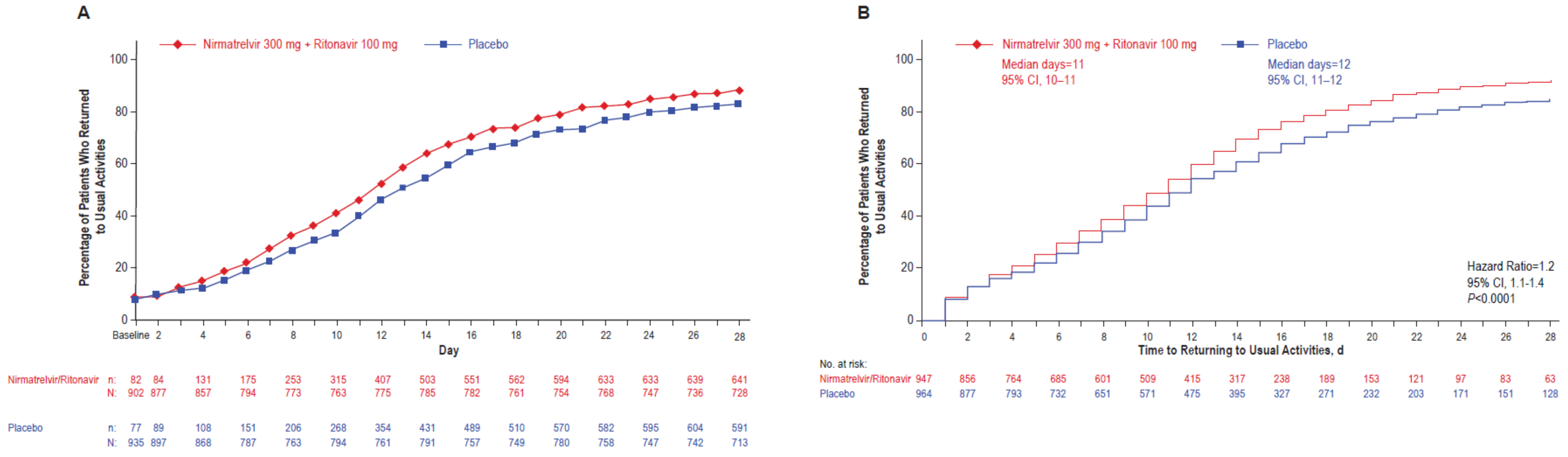

Supplementary Figure 3. Modified intent-to-treat population 1: patient perceptions of daily COVID-19 symptom severity for 28 days according to the Global Impressions Questionnaire. (A) Percentage of patients with severe COVID-19 symptoms, (B) time to achieving sustained resolution of moderate to severe overall symptoms, (C) time to achieving sustained resolution of any overall symptoms, (D) time to achieving sustained alleviation of any overall symptoms, and (E) percentage of patients reporting worsening of symptoms. The modified intent-to-treat 1 population included all participants who were randomly assigned to study intervention, received ≥1 dose of study intervention, at baseline did not receive nor were expected to receive COVID-19 therapeutic monoclonal antibody treatment, and were treated within ≤5 days of COVID-19 onset. n=number of patients with event; N=number of patients with nonmissing data.

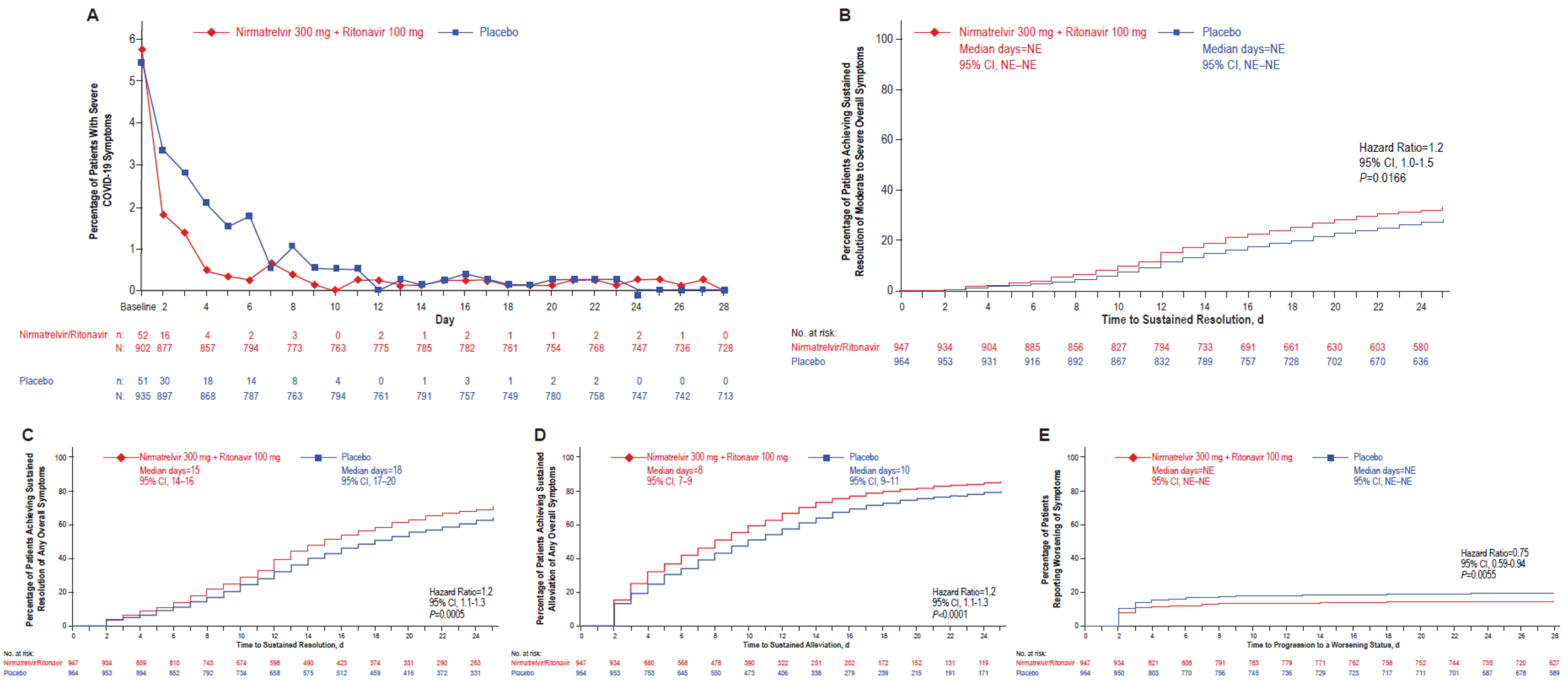

Supplement: ofaf449_Supplementary_Data [file ofaf449_supplementary_data.pdf]
